# Conquering the inner couch potato: precommitment is an effective strategy to enhance motivation for effortful actions

**DOI:** 10.1098/rstb.2018.0131

**Published:** 2018-12-31

**Authors:** Bettina Studer, Carolin Koch, Stefan Knecht, Tobias Kalenscher

**Affiliations:** 1 Institute of Clinical Neuroscience and Medical Psychology, Medical Faculty, University of Düsseldorf, Moorenstrasse 5, 40225 Düsseldorf, Germany; 2 Mauritius Hospital Meerbusch, Strümperstraße 111, 40670 Meerbusch, Germany; 3 Comparative Psychology, Institute of Experimental Psychology, University of Düsseldorf, Universitätsstrasse 1, 40225 Düsseldorf, Germany

**Keywords:** self-control, opportunity cost, effort discounting, exercise, temporal discounting, motivation

## Abstract

Letting effort-free gratification derail us from effort-requiring goals is one reason why we fail to realize health-relevant intentions like ‘exercise regularly’. We tested the effectiveness of the self-control strategy precommitment in such effort-related conflicts, using a novel laboratory choice paradigm, where participants could precommit to an effort-requiring large reward by pre-eliminating an effort-free small reward from their choice set. Our participants used precommitment frequently and effectively, such that they reached effort-requiring large rewards more often. Using computational modelling and Bayesian model comparisons, we assessed whether participants employed precommitment to avoid anticipated willpower failures (i.e. as a self-regulatory measure) or to maximize their motivation to choose the effort-requiring option (i.e. as a self-motivational measure). Observed choices and precommitment decisions were consistent with the motivation maximization hypothesis, but not the willpower hypothesis. Our findings show that offering precommitment is effective in helping individuals optimize their motivation and choice behaviour and thereby achieve effort-requiring goals, and strongly encourage application of precommitment schemes in exercise and rehabilitation interventions.

This article is part of the theme issue ‘Risk taking and impulsive behaviour: fundamental discoveries, theoretical perspectives and clinical implications’.

## 1. Introduction

Failures to put effort-requiring health-relevant intentions like ‘exercise twice a week’ or ‘use the stairs’ into action are experienced by most people. A third of us are physically inactive [1], we do not execute our workout intentions [2], and even motivated stroke patients suffering from disabling motor impairments conduct less rehabilitative training than intended [3]. Why does this happen and how could it be overcome? Models of exercise behaviour posit that such intention-behaviour gaps are caused by failures in self-regulation and assume motivation to exercise to be stable (e.g. [4–6]). By contrast, neuroeconomic models highlight the reference-dependent nature of motivation and posit that the probability of choosing an activity is not only determined by its subjective value but also by that of simultaneously available alternatives, referred to as ‘opportunity costs’ (see [7–9] for reviews). Here, failures to execute intentions occur when opportunity costs outweigh the subjective value of the target activity. Moreover, effort requirements and delay to reward receipt are considered costs and diminish the subjective value of a reward (e.g. [10–13]). This can help explain why actions such as watching TV, which require no effort and lead to instant gratification, are chosen over effort-requiring physical exercise, even when the latter ultimately conveys greater reward. Our preference for effort-free or instant rewards can be overcome through self-regulatory measures, such as active inhibition of choosing instant/effort-free gratification—a cognitive process termed ‘willpower’ [14]. An alternative strategy, named ‘precommitment’, entails strategic modulations of one's (future) choice set, such as removing tempting, but ultimately inferior, choice options, adding unattractive consequences to such alternatives or inflating their costs [15–18]. Precommitment schemes were shown to raise retirement saving rates [19], chances of smoking cessation [20], healthy food shopping [21] and choices of delayed rewards over instant gratification [22]. But, it remains unexplored whether precommitment can also help to increase choices of effortful actions over effortless gratification, even though this would be highly relevant for interventions targeting physical inactivity and sedentary lifestyle in the still healthy and patient populations (see [23]). Our goal was to test the effectiveness of precommitment in the context of effort-requiring rewards and thereby provide an empirical cornerstone for clinical application of precommitment schemes in rehabilitation and exercise interventions.

We administered a novel effort-based decision-making paradigm (henceforth termed ‘effort task’) to healthy middle-aged men (*n* = 59), in which they made choices between an effortless small reward (SR) and a larger reward (LR) that required physical effort (squeezes on a hand dynamometer). Erotic images served as rewards, with the image set personalized for every participant. Two trial types were contrasted: *standard trials*, where participants could still abandon an initially selected effort-requiring LR for the effortless SR throughout the (post-choice) effort-execution phase, and *precommitment trials*, where subjects could voluntarily precommit to the effort-requiring LR by removing the effortless SR option ahead of the choice. Effectiveness of precommitment was quantified through the proportion of achieved large rewards in precommitment compared to standard trials. We also administered an intertemporal choice paradigm (‘delay task’, modified from Crockett *et al*. [22]), in which participants made choices between an immediate SR and an LR that required waiting for. This task again contrasted standard trials, where participants could still abandon an initially selected delayed LR for the instant SR throughout the (post-choice) waiting period, and precommitment trials, where subjects could voluntarily precommit to the waiting-requiring LR by removing the effortless SR option ahead of the choice.

Our second goal was to examine to what purpose participants employed precommitment in the two tasks. Using computational modelling of participants' choices and precommitment decisions together with Bayesian model comparison techniques, we assessed two competing hypotheses. The ‘willpower hypothesis’ postulates that precommitment is employed to prevent anticipated failures in willpower, i.e. as a self-regulatory measure (e.g. [15,22,24,25]). The ‘motivation maximization hypothesis' builds on the premise that the motivation for a target activity (in our tasks, choosing the effort-requiring/delayed LR option) is diminished by opportunity costs and posits that precommitment is employed to maximize ones (net) motivation by reducing these opportunity costs, i.e. as a self-motivational measure [9]. These hypotheses make distinct predictions about when precommitment is employed. The willpower hypothesis predicts that subjects will choose to precommit when willpower is (assumingly) not sufficient to ensure achievement of the ultimately greater, but effort-requiring or delayed large reward. The motivation maximization hypothesis predicts that precommitment is used when the individual has a target (i.e. preferred) action, but the (net) motivation for this action is reduced by opportunity costs. We put these two competing theoretical accounts to the test by assessing whether observed choices and precommitment decisions in our tasks are better explained by computational models derived from the motivation maximization hypothesis or from the willpower hypothesis.

## 2. Methods

### (a) Participants and procedures

Fifty-nine heterosexual males (aged 18–77, mean = 37.44, s.d. = 18.58; years of education: mean = 16.61, s.d. = 3.71) participated in this study, which was approved by the ethics committee of the Institute of Experimental Psychology, University of Düsseldorf. The sample size was determined through *a priori* calculation using G*Power 3 [26]: based on Crockett *et al.* [22], we expected precommitment to have a moderate effect (*d*_z_ = 0.5) upon our main outcome measure proportion of large rewards achieved, resulting in a critical sample size of *n* = 54 (with power = 0.95). To assure this critical sample size was met even in the case of potential drop-outs, we raised the target sample size by 10% to *n* = 59. Subjects underwent a single testing session lasting approximately 90 min. First, they rated 400 images of women in lingerie/swimwear (300 × 380 pixels, 24-bit colour depth) on a scale of 0–10. The ratings were then used to construct an individualized reward image set for each subject, through the procedure established in Crockett *et al*. [22]. In short, images rated as 0 (not enjoyable) or 1 (neutral) were discarded, images rated higher than the subjects’ median rating were designated as large rewards and images rated below the subjects' median as small rewards. Next, the participant's maximum handgrip strength was determined (see electronic supplementary material for details). Then, the two main experimental tasks were administered in randomized order.

### (b) Main experimental tasks

#### (i) Effort task

**Figure 1.**
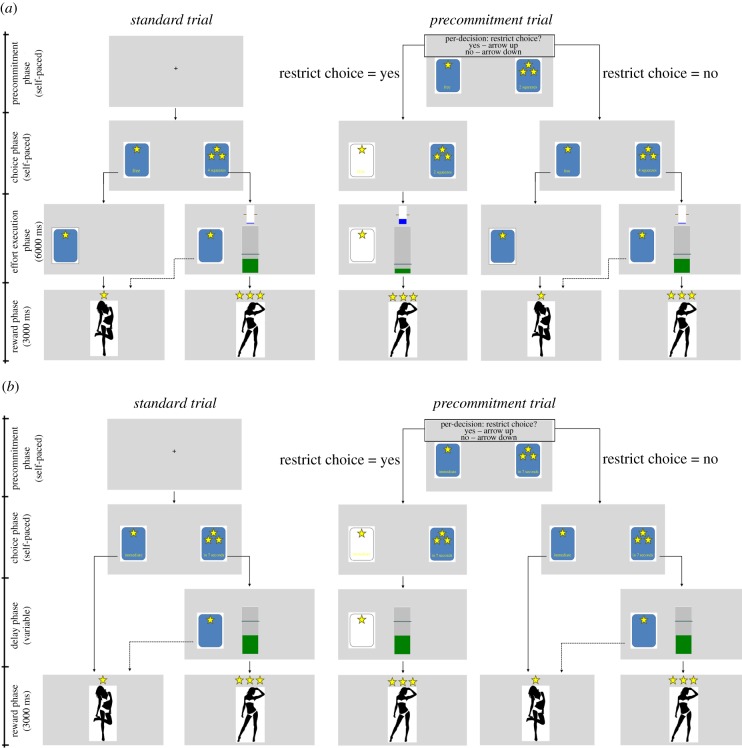
Experimental tasks. In both tasks, subjects chose between a free small reward (SR) option, represented by a 1-star card, and a costly large reward (LR) option, represented by a 3-star card. In the effort task (*a*), the LR required an indicated number of squeezes (2, 4 or 6) on a hand dynamometer exceeding 50% of the individuals' max. strength. The SR required no effort production. In the delay task (*b*), the LR required waiting an indicated number of seconds (4, 7 or 10), whereas the SR was provided immediately. Blue cards indicate options available for selection, white cards indicate unavailable SRs. The left panels show the possible courses of a standard trial. Note that, following a choice of the LR option, the SR option remained available throughout the effort execution/delay phase and subjects could still abandon the LR option at any time. The right panels show the possible courses of a precommitment trial. Here, subjects first decided whether to precommit to the effort-requiring/delayed LR by making the SR unavailable for the current trial (see right panel, left column). If they decided against this voluntary restriction of their choice set, the trial continued identically to the standard trial (right panel, right column). The frame around the text displayed during the pre-choice precommitment phase is for illustration purposes only and was not present during the task. See main text for further explanations.

In this paradigm, participants made choices between an effortless SR and an LR that required a variable number of squeezes on a hand-held dynamometer (three levels: effort_LR_ = 2, 4 or 6 squeezes). A squeeze was defined as a short contraction of the dynamometer at equal to or greater than 50% of the individuals' maximal strength followed by complete release. Individualized force levels were used in order to match subjective effort levels across participants. The task contrasted standard and precommitment trials. Standard trials consisted of an initial choice phase, an effort-execution phase (6000 ms) and a reward delivery phase (3000 ms; figure 1*a*, left panel). During the choice phase, two cards were presented, a 1-star card representing the SR and a 3-star card representing the LR. Effort requirements were indicated on the cards, with the SR option (1-star card) always offered for free, i.e. requiring zero squeezes. The SR and LR options were randomly displayed on the left or right side of the screen. Once the participant indicated their choice (with a left or right button-press via keyboard), the effort-execution phase started. Duration of the execution phase was fixed to 6 s in all trials, so as to keep delay to reward receipt constant and thereby avoid a time confound due to longer execution period after LR choices. If the subject chose the effort-requiring LR option, two dynamic bars were displayed during the effort-execution phase: a centrally displayed green bar counting the executed squeezes with the required number of squeezes indicated by a target line, and a smaller blue bar visualizing the currently applied force on the dynamometer and the 50% maximum force level that had to be exceeded for a squeeze to be counted. Crucially, the effort-free SR option remained visible on screen throughout this execution phase, and participants were free to abandon effort production and choose the SR instead at any time. In the reward delivery phase, the chosen reward was presented. Precommitment trials differed from standard trials in that participants could exclude the effort-free SR option for the trial ahead of the main choice period (see figure 1*a*, right panel). If participants decided not to precommit, the trial continued identically to a standard trial, and the effort-free SR option was available during the choice and effort-execution phases. Note that receipt of the LR was always contingent upon having completed the required number of squeezes during the effort production phase; if the subject failed to do so upon reaching the reward delivery phase (and had not already opted-out of the LR option), the message ‘required number of squeezes not achieved’ was displayed and a SR was delivered.^1^ Trials were separated by a 2000 ms inter-trial interval (ITI) during which a fixation cross was displayed. Subjects were administered 30 precommitment and 30 standard trials, with each of the three levels of required effort for the LR appearing ten times per trial type, in randomized order. The proportion of achieved large rewards served as the main outcome measure.

#### (ii) Delay task

The delay task was adapted from Crockett *et al*. [22] and was structurally analogous to the effort task. On each trial, participants chose between an immediate SR option and an LR that was delivered only after a variable waiting period (three levels: delay_LR_ = 4, 7 or 10 s, figure 1*b*). Again, the task contrasted standard and precommitment trials, each of which contained a choice phase (self-paced), a waiting phase (variable length, 0–10 s), a reward delivery phase (3000 ms) and an ITI (2000 ms). In the choice phase, two cards were again presented, a 1-star card representing the immediate SR option and a 3-stars card representing the delayed LR option, with the required waiting time indicated on the card. If the subject selected the immediate SR option (by a corresponding button press), they entered the reward delivery phase immediately. If the subject selected the delayed LR, a dynamic bar counting the seconds up to a vertical target line was displayed during the waiting phase. Crucially, the immediate SR reward option remained visible on screen throughout the delay phase, and participants were free to give up waiting for the LR and choose the SR instead at any time. In precommitment trials, participants could exclude the immediate SR option for the given trial prior to the main choice phase and precommit to the delayed LR (see figure 1*b*, right panel). If participants decided not to precommit, the trial continued identically to a standard trial. Subjects were administered 30 precommitment trials and 30 standard trials, with each of the three levels of required waiting times for the LR appearing ten times in each trial type, in randomized order. Again, the proportion of achieved large rewards served as the main outcome measure.

### (c) Model-free data analyses

Data from one subject were lost due to technical problems during data collection, thus the sample size in statistical analyses was *n* = 58. We first explored whether participants' choices were sensitive to the effort/waiting requirements of the LR option and whether large rewards were achieved more frequently in precommitment trials, i.e. whether offering a precommitment option was effective. For each participant, we extracted the trial-by-trial outcome variable ‘Achievement of LR’ (binary, 1-yes, 0-no) and entered this outcome variable into a repeated measures logistic regression model with the categorical predictors ‘Trial Type’ (precommitment versus standard) and ‘Waiting/Effort Requirement of the LR’. Separate models were calculated for the two experimental tasks, and choice of working correlation matrix was based on the quasi-likelihood under independence model criterion (QIC) as an index of model fit. Wald *χ*^2^ statistics for model effects and predicted probabilities are reported. We also tested the addition of a ‘Trial Type×Waiting/Effort Requirement Interaction’ predictor to these regression models, but since this resulted in poorer model fits (higher QIC) and non-significant interaction effects, these extended models were discarded (see electronic supplementary material for details on this and on a second alternative model testing for potential effects of time-on-task). Additionally, we examined rates of opt-outs (i.e. instances where an initially chosen LR was abandoned during the effort execution/delay phase) in standard trials and rates of precommitment in precommitment trials. Complimentary analyses of response times are reported in the electronic supplementary material.

### (d) Computational models

The model-free analyses revealed that precommitment was both frequently used and effective in enhancing the proportion of achieved large rewards. Next, we turned to the question of to which purpose participants used precommitment: to avoid anticipated failures in willpower (willpower hypothesis) or to maximize their motivation to choose the effortful/delayed LR by extinguishing opportunity costs (motivation maximization hypothesis). To answer this question, we formulated two computational models for each hypothesis, one that models participants' choices between the effort-requiring/delayed LR option versus the effort-free/immediate SR option without precommitment (‘choice model’) and one that models participants' decisions to precommit to the effort/waiting-requiring LR (‘precommitment model'; see table 1 for an overview and figure S2 in the electronic supplementary material for a visualization of the choice model parameters). The choice models of both hypotheses posit that choices are determined by the difference in the subjective values of the two choice options and assume hyperbolic discounting of rewards by required effort and waiting/delay [10,13,27–29]. The choice model of the motivation maximization hypothesis (‘MM choice model’) defines the net motivational value of choosing the effort-/waiting-requiring LR option (Δ*V*) as the difference between its subjective value and its opportunity costs, i.e. the subjective value of the effort-free/immediate SL alternative, and was formulated as:

$$\;\Delta V=SV_{\mathrm{LR}}-OC_{\mathrm{LR}}\;(\mathrm{MM}\;\mathrm{choice}\;\mathrm{model}),$$

$$\;OC_{\mathrm{LR}}=SV_{\mathrm{SR}}=M_{\mathrm{SR}}\;$$

$$\begin{array}{cc} \mathrm{for}\;\mathrm{effort}\;\mathrm{task}\; & SV_{\mathrm{LR}}=\;\frac{M_{\mathrm{LR}}}{\left(1+\varepsilon E_{\mathrm{LR}}\right)}\;\\ \mathrm{for}\;\mathrm{delay}\;\mathrm{task}\; & SV_{\mathrm{LR}}=\;\frac{M_{\mathrm{LR}}}{\left(1+\kappa D_{\mathrm{LR}}\right)},\; \end{array}$$

where *SV*_LR_ and *SV*_SR_ are the subjective values of the LR and SR options, *OC*_LR_ is the opportunity cost of choosing the LR option, *M*_LR_ and *M*_SR_ are the subject-specific average ratings for the LR and SR images (i.e. the reward magnitude), *E*_LR_ and *D*_LR_ are the required effort and waiting period of the LR, respectively, *ɛ* and *κ* are estimated parameters reflecting the steepness of effort discounting and of delay discounting, respectively, and Δ*V* is the net motivational value of the effort-/waiting-requiring LR option.

**Table 1.** Overview of the main computational models. N.B. The WP choice model was additionally fitted to achieved rewards on standard trials and precommitment trials in which subjects *rejected* precommitment to estimate *ι*_rewards._

choice models					
description models of participants’ choices of the LR without precommitment			fitted to choices on standard trials and precommitment trials in which subjects rejected precommitment		
excluded data: precommitment trials in which subjects rejected precommitment					
effort task	model parameters		*n* trials per subject		
	MM choice model	WP choice model	mean	s.e.m.	range
	*ɛ*, *γ*	*ɛ*, *γ*, *ι*_choice_	44.2	1.59	[30,60]
					
delay task	model parameters		*n* trials per subject		
	MM choice model	WP choice model	mean	s.e.m.	range
	*κ*, *γ*	*κ*, *γ*, *ι*_choice_	45.1	1.57	[30,60]

precommitment models					
description models of participants' precommitment decisions			fitted to precommitment decisions in precommitment trials		
excluded data: standard trials					
effort task	model parameters				
	MM precom model	WP precom model	*n* trials per subject		
	*θ*, *b*	*θ*, *b*	15		
delay task	model parameters				
	MM precom model	WP precom model	*n* trials per subject		
	*θ*, *b*	*θ*, *b*	15		

The choice model of the willpower hypothesis (WP choice model) additionally assumes application of willpower, in the form of active suppression of the true subjective value of the tempting effort-free/immediate SR option during choice, and was formulated as:

$$\;\Delta V=SV_{\mathrm{LR}}-\iota SV_{\mathrm{SR}}\;(\mathrm{WP}\;\mathrm{choice}\;\mathrm{model}),$$

$$\;SV_{\mathrm{SR}}=M_{\mathrm{SR}}\;$$

$$\begin{array}{cc} \mathrm{for}\;\mathrm{effort}\;\mathrm{task}\; & SV_{\mathrm{LR}}=\;\frac{M_{\mathrm{LR}}}{\left(1+\varepsilon E_{\mathrm{LR}}\right)}\;\\ \mathrm{for}\;\;\mathrm{delay}\;\mathrm{task}\; & SV_{\mathrm{LR}}=\;\frac{M_{\mathrm{LR}}}{\left(1+\kappa D_{\mathrm{LR}}\right)},\; \end{array}$$

with the estimated parameter *ι* representing the wilful suppression of the true subjective value of the effort-free/immediate SR during choice. This willpower parameter was restricted to 0–1, with *ι* = 1 signifying no suppression of the subjective value of the SR and *ι* = 0 signifying complete suppression.

Both choice models were calculated separately for each experimental task by fitting them to subjects' trial-by-trial responses during the choice phase without precommitment (i.e. on standard trials and trials where precommitment was rejected^2^) using a softmax function-based prediction:

$$P(\mathrm{Choice}\;\mathrm{LR})=\frac{1}{\left(1+e^{-\gamma \Delta V}\right)},$$

where *γ* is a subject-specific inverse temperature parameter that characterizes the sensitivity of choices to ΔV, and P(Choice LR) is the probability of selecting the effort-/waiting-requiring LR option.

The willpower hypothesis further posits that the wilful suppression of the SR will sometimes fail, leading to a direct choice of the SR option or to an opt-out of an initial LR choice during the delay/effort-execution period. To test this prediction, we also fitted the WP choice model to subjects' actual achieved rewards (on standard trials and trials where precommitment was rejected) using

$$P(\mathrm{LR}\;\mathrm{Achievement})=\frac{1}{\left(1+e^{-\gamma \Delta V}\right)},$$

and tested whether estimates of the willpower *ι* obtained when fitting the WP choice model to choices (henceforth labelled *ι*_choice)_ were significantly smaller (representing stronger suppression) than estimates of *ι* obtained when fitting the WP choice model to achieved rewards (henceforth labelled *ι*_reward_) through a paired *t*-test.

The WP and MM precommitment models used the subject-specific value estimates derived from the respective choice model to describe precommitment decisions. The willpower hypothesis posits that precommitment is employed when wilful suppression of the subjective value of the SR is not enough to ensure achievement of the LR (i.e. when *ι*_reward_*SV*_SR_ > *SV*_LR_). Therefore, the precommitment model of the willpower hypothesis formulates the value of precommitment (*V*_Precom_) as

$$V_{\mathrm{Precom}}=\iota_{\mathrm{reward}}SV_{\mathrm{SR}}-SV_{\mathrm{LR}}\;(\mathrm{WP}\;\mathrm{precommitment}\;\mathrm{model}),$$

where *ι*_reward_, *SV*_SR_ and *SV*_LR_ are the willpower parameter and the subjective values of the SR and the LR obtained when fitting the willpower choice model to actually achieved rewards without precommitment, respectively.

Meanwhile, the motivation maximization hypothesis posits that precommitment is used to maximize the net motivational value for choosing the *preferred* effort/waiting-requiring LR option by eliminating opportunity costs, that is to say, employed when the subjective value of the effort/waiting-requiring LR option is higher than of the SR option. Thus, the precommitment model of the motivation maximization hypothesis formulates *V*_Precom_ as

$$V_{\mathrm{Precom}}=SV_{\mathrm{LR}}-SV_{\mathrm{SR}}\;(\mathrm{MM}\;\mathrm{precommitment}\;\mathrm{model}),$$

where *SV*_SR_ and *SV*_LR_ are subjective values of the SR and the LR obtained when fitting the MM choice model to participants' choices without precommitment.

Both precommitment models were calculated separately for each experimental task by fitting them to subjects' trial-by-trial precommitment decisions using

$$P(\mathrm{Precommitment})=\;\frac{1}{\left(1+e^{-(\theta \mathrm{VPrecom}+b)}\right)},$$

where *θ* is a subject-specific inverse temperature parameter that characterizes how strongly *V*_Precom_ influences the precommitment decision, P(Precommitment) is the probability of precommitment to the effort-/waiting-requiring LR, and *b* describes the subjects' general propensity to use precommit, with *b* < 0 indicating a bias towards and *b* > 0 indicating a bias away from precommitment.^3^

For all models, subject-specific parameters were optimized across trials using nonlinear optimization implemented in Matlab (MathWorks, Inc.) for maximum-likelihood estimation. Estimates were found to be reliable and were confirmed with multiple random starts of optimization. We used Bayesian comparison techniques [30,31], specifically the Bayesian information criterion (BIC), to compare the fits of our models to the observed data. The BIC follows the principle of parsimony, which states that the simplest model that can explain the data is the best, and protects against overfitting (i.e. misattributing noise as signal). Thus, the BIC gauges goodness-of-fit, i.e. how well the model can account for the observed data, against model complexity (see [32] for more extensive explanation of the importance of this trade-off). We computed a group BIC score for each model by summing up calculated BIC scores for each individual. A better (corrected) fit is represented by a smaller (group) BIC score, and BIC differences greater than 2 are considered positive evidence [30]. In addition, we report the average McFadden's pseudoR^2^ for each model. PseudoR^2^ is an uncorrected measure of model fit, ranging between 0 and 1, which expresses how much better the model predicts the data compared to a null model (chance prediction). Values of 0.2 or higher indicate an excellent fit [33]. Considering pseudoR^2^s alongside BIC scores allowed us to determine whether a model's superiority as established by a lower BIC score was due to better prediction of the observed data, better parsimony, or both. This was particularly informative in the case of the comparison of the two choice models because the MM choice model is by design a less complex model than the WP choice model (with the additional *i* parameter). When examining the MM and WP precommitment models, we also compared the obtained *θ* parameters, expressing how sensitive participants' precommitment decisions were to the hypothesized value of precommitment (*V*_precom_), through a paired *t*-test.

In addition, we ran parameter recovery simulations to check whether the parameters of our models can be reliably determined within the observed range of parameter combinations (for details, see electronic supplementary material). These revealed that the *ι* parameter of the WP choice models could not be reliably estimated if a subject applied no effort or delay discounting at all (*ε* or *κ* ≈ 0), which could be recognized by a complete lack of variance in their LR choices (LR option chosen in all trials). Removing such participants from the choice model comparisons did not lead to qualitative differences in the results of the WP choice model comparisons (see electronic supplementary material for a full report).

In a final step, we examined individual differences in participants’ general tendency to use precommitment, quantified by the subject-specific *b* parameter obtain from the (winning) MM precommitment model. We then tested whether participants' propensity to precommit (model-estimated *b*s) correlated across the two tasks, using Spearman rank correlation analysis. Additionally, we tested whether the subject-specific effectiveness of precommitment, quantified by the relative increase in achieved LR in precommitment compared to standard trials, correlated across the two tasks.

All statistical tests were carried out in SPSS (v22, IBM) and are reported two-tailed with *α* set to 0.05.

## 3. Results

### (a) Effort task

**Figure 2.**
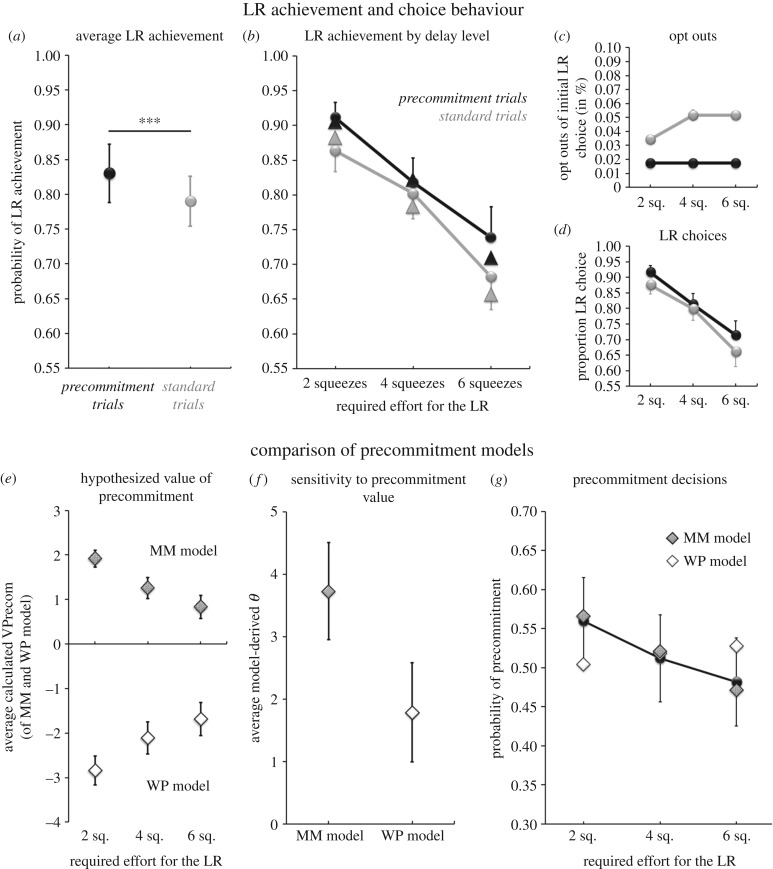
Effort task. (*a,b*) Effectiveness of precommitment: proportion of achieved effort-requiring LRs was higher in precommitment trials (black) than in standard trials (grey; *a*) and declined with increasing effort requirements (*b*; triangles, predicted probability; circles, observed data). (*c,d*) Choice behaviour: opt outs of an initial LR choice during the waiting period were rare (*c*) and proportion of LR choices (without or with previous precommitment) showed a similar pattern to LR achievement. (*e*–*g*) Comparison of the MM and WP precommitment models. (*e*) Average value of precommitment to the LR option at each waiting requirement level as determined by the MM and WP model. (*f*) The average obtained *θ* value reflecting sensitivity of participants’ precommitment decisions to the model-predicted value of precommitment was numerically higher for the MM model than for the WP model, and this difference became statistically significant (*p* < 0.5) when subjects with unreliable parameter estimation were excluded (see main text for details). (*g*) Observed precommitment rates (black circles) closely aligned with the predicted probabilities of precommitment by the MM precommitment model (grey diamonds), but showed the opposite pattern than that predicted by the WP precommitment model (white diamonds). Error bars represent s.e.m.; *** denotes a significant effect with *p* ≤ 0.001.

As expected, participants’ choices were sensitive to the effort requirements of the LR option, with odds of achieving the LR decreasing with increasing levels of required effort, Wald $\chi^{2}(2)=25.31$, *p* < 0.001. Across both trial types, probability of achieving the LR was determined as 0.89 when it required 2 squeezes, 0.80 when it required 4 squeezes and 0.68 when it required 6 squeezes (s.e.m. = 0.024, 0.035, 0.046, respectively). Moreover, precommitment was both frequently used and effective: on average, participants precommitted to the effort-requiring LR option in 52% of precommitment trials (s.e.m. = 5%), and—as a consequence—rates of achieving the LR were modestly but robustly higher in precommitment trials, Wald $\chi^{2}(1)=11.638$, *p* = 0.001, with an average predicted probability of achieving the LR of 0.83 in precommitment trials (s.e.m. = 0.042) versus of 0.79 in standard trials (s.e.m. = 0.036; figure 2*a*). Predicted and observed proportions of LR achievement at each level of effort_LR_ and trial type are displayed in figure 2*b*. Intriguingly, the frequent and effective use of precommitment occurred despite very low opt-out rates: on average, opt-outs were observed in only 0.5% of standard trials and 88% of participants never abandoned a chosen effort-requiring LR option during the effort-execution phase (figure 2*c*). In summary, the model-free analyses demonstrated that participants used precommitment frequently and effectively on the effort task.

#### (i) Purpose of precommitment in the effort task (model-based analyses)

We first tested whether choices without precommitment (i.e. on standard trials and trials where the participant decided against precommitment) were better explained by the willpower (WP) or the motivation maximization (MM) choice model. The MM choice model strongly outperformed the WP choice model in the Bayesian model comparison, both on the group level (BIC_MM model_ = 1975, BIC_WP model_ = 2189) and for each individual participant (Δ individual BIC scores: mean = −3.69, range [−3.14, −4.09], see electronic supplementary material, figure S2). The average pseudoR^2^s of the two models were identical and showed excellent fit (both models: mean = 0.60, s.e.m. = 0.05). Thus, the observed choices of participants without precommitment were equally well predicted by both models, but more parsimoniously explained by the less complex MM model (percentage of choices correctly predicted: mean = 0.86, s.e.m. = 0.019, see table 2 for parameter estimates). Furthermore, WP model-derived estimates of *ι*_reward_ (mean = 0.50, s.e.m. = 0.044) were not systematically smaller than estimates of *ι*_choice_ (mean = 0.49, s.e.m. = 0.045, *t*_57_ = −0.170, *p* = 0.865), contrary to the assumption of the willpower hypothesis that wilful suppression of the SR would sometimes fail during the effort execution period, but consistent with the observation that opt-outs of initial LR choices were very rare. In summary, the choice model comparison showed that the willpower hypothesiś assumption of wilful suppression of the effort-free SR during choice is not needed to explain observed choices without precommitment. Rather, participants' choices between the effort-free SR and the effort-requiring LR option can be equally well and more parsimoniously explained by the motivation maximization model.

**Table 2.** Estimated parameters of the MM choice model.

	mean	s.e.m.	range
effort task			
*ɛ*	0.206	0.036	[0, 1]
*γ*	6.59	0.832	[0, 21.25]
delay task			
*κ*	0.155	0.032	[0, 1]
*γ*	20.72	2.99	[13.30, 67.20]

Next, we assessed which hypothesis could better explain participants’ precommitment decisions. The MM precommitment model strongly outperformed the WP precommitment model in Bayesian model comparison (BIC_MM model_ = 1249, BIC_WP model_ = 1348) and also had a significantly higher average pseudoR^2^ (MM model = 0.64, WP model = 0.60, mean difference = 0.04, *t*_57_ = 3.181, *p* = 0.002). Figure 2*e* and *g* display the model-determined average values of precommitment and observed and predicted precommitment probabilities for each effort requirement level of the LR, respectively. Moreover, *θ* estimates obtained for the MM model (mean = 3.73) were larger than those obtained for the WP model (mean = 1.79, mean difference = 1.94, *t*_57_ = 1.60, *p* = 0.12; figure 2*f*), and this difference became statistically significant after exclusion of participants who either always (*n* = 11) or never (*n* = 10) accepted precommitment choices (MM model mean = 2.27, WP model mean = 0.421, mean difference = 1.85, *t*_36_ = 2.03, *p* = 0.049). This result signifies that participants' precommitment choices were better explained by the value of precommitment determined by the MM precommitment model than by that determined by the WP precommitment model. In summary, comparison of the precommitment models showed that the motivation maximization hypothesis better predicts observed precommitment decisions on our effort task than the willpower hypothesis, indicating that subjects used precommitment to increase their net motivation for the effort-requiring LR option by eliminating opportunity costs, rather than to overcome anticipated failures in willpower.

### (b) Delay task

**Figure 3.**
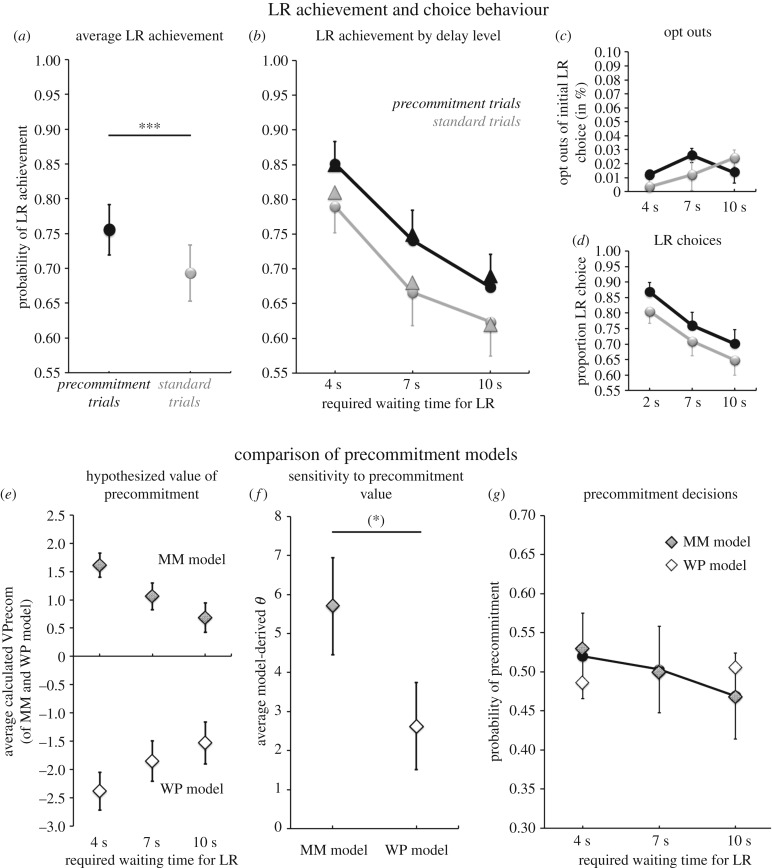
Delay task. (*a,b*) Effectiveness of precommitment*:* proportion of achieved delayed LRs was higher in precommitment trials (black) than in standard trials (grey; *a*) and declined with increasing waiting requirements of the LR (*b*; triangles, predicted probability; circles, observed probability of LR achievement). (*c,d*) Choice behaviour: opt outs of an initial LR choice during the waiting period were rare (*c*) and proportion of LR choices (without or with previous precommitment) showed a similar pattern to LR achievement. (*e*–*g*) Comparison of the MM and WP precommitment models. (*e*) Average value of precommitment to the LR option at each waiting requirement level as determined by the MM and WP model. (*f*) The average obtained *θ* value reflecting sensitivity of participants' precommitment decisions to the model-predicted value of precommitment was higher for the MM model than for the WP model. (*g*) Observed precommitment rates (black circles, partially hidden behind grey diamonds) closely aligned with the predicted probabilities of precommitment by the MM precommitment model (grey diamonds), but showed the opposite pattern than that predicted by the WP precommitment model (white diamonds). Error bars represent s.e.m.; *** denotes a significant effect with *p* ≤ 0.001, (*) denotes a tentative effect with *p* < 0.1 (two-tailed).

As expected, participants’ choices on the delay task were sensitive to the required waiting time for the LR option, with odds of achieving the LR decreasing with longer waiting requirements, Wald $\chi^{2}(2)=19.44$, *p* < 0.001. Across both trial types, the probability of achieving the LR was predicted as 0.83 for delay_LR_ = 4 s, 0.72 for delay_LR_ = 7 s and 0.66 for delay_LR_ = 10 s (s.e.m. = 0.033, 0.044, 0.047, respectively). On average, participants decided to precommit to the waiting-requiring LR option in 49% of precommitment trials (s.d. = 5%). And, offering precommitment was effective: participants were more likely to achieve the LR in precommitment trials, Wald $\chi^{2}(1)=17.922$, *p* < 0.001, with an average predicted probability of 0.77 in precommitment trials (s.e.m. = 0.042) versus 0.71 in standard trials (s.e.m. = 0.036; figure 3*a*). Predicted and observed proportions of achieving the LR at each delay level and trial type are displayed in figure 3*b*. And again, this frequent use of precommitment occurred despite opt-out rates in standard trials being low: opt-outs were observed only in 1.7% of standard trials on average (figure 3*c*) and 76% of participants never abandoned a chosen delayed LR option during the waiting phase. In summary, the model-free analyses again demonstrated that participants used precommitment frequently and effectively.

#### (i) Purpose of precommitment in the delay task (model-based analyses)

As in the case of the effort task, we first tested whether participants' choices between the immediate SR and the waiting-requiring LR without precommitment (i.e. on standard trials and trials where participants decided against precommitment) were better explained by WP choice model or the MM choice model. The MM choice model again strongly outperformed the WP choice model in Bayesian model comparison, both on the group level (BIC_MM model_ = 2044, BIC_WP model_ = 2256) and for every individual participant (difference in individual BIC scores: mean = −3.67, range [−2.80, −4.09]; see electronic supplementary material, figure S3). The average pseudoR^2^s of the two models were identical and showed excellent fit (both models: mean = 0.59, s.e.m. = 0.05), but, as in the effort task, the less complex MM choice model constitutes the more parsimonious explanation of participants’ choices (percentage of choices correctly predicted: mean = 0.87, s.e.m. = 0.018; figure 3*c* for estimated Δ*V* at each delay_LR_ level and table 3 for parameter estimates). And again, no significant differences between the willpower parameters *ι*_reward_ (mean = 0.47, s.e.m. = 0.046) and *ι*_choice_ (mean = 0.49, s.e.m. = 0.045), obtained by fitting the WP model to choices and rewards achieved, respectively, were found (*t*_57_ = −0.730, *p* = 0.469), in contrary to the assumptions of the willpower hypothesis. In summary, the WP hypothesis' assumption of wilful suppression is unnecessary to explain participants’ choices between the immediate SR and waiting-requiring LR (without precommitment). Rather, the observed choices could be equally well explained by the more parsimonious MM model, which formulates these choices as purely value-based decisions.

**Table 3.** Estimated parameters of the MM precommitment model.

	mean	s.e.m.	range
effort task			
* θ*	3.73	0.776	[0, 26]
* b*	−1.46	0.388	[−5, 5]
delay task			
* θ*	5.70	1.25	[0, 40]
* b*	−1.10	0.382	[−5, 5]

Next, we assessed which hypothesis could better explain participants' precommitment decisions on the delay task. As in the effort task, the MM precommitment model again outperformed the WP precommitment model in Bayesian model comparison (BIC_MM model_ = 1328, BIC_WP model_ = 1378) and also had a modestly, but robustly, higher average pseudoR^2^ (MM model mean = 0.61, WP model mean = 0.59, mean difference = 0.021, *t*_57_ = 2.076, *p* = 0.04). Average values of precommitment according to each precommitment model are displayed in figure 3*e*, observed and predicted precommitment probabilities at each required waiting level for the LR are in figure 3*g*. Moreover, the *θ* estimate of the MM model (mean = 5.70) was tentatively higher than that of the WP model (mean = 2.63), meaning that participants precommitment choices were more closely related to the MM model's value of precommitment than to that of the WP model (mean difference = 3.07, *t*_57_ = 1.69, *p* = 0.09; figure 3*f*). In summary, the MM model better explained subjects’ precommitment decisions on the delay task than the WP model, indicating that participants used precommitment to enhance their net motivation to choose the waiting-requiring LR option by eliminating opportunity costs, rather than to prevent anticipated failures in wilful suppression of the instant SR option.

### (c) Individual differences in propensity to precommit

Participants’ propensity to precommit (quantified by *b* estimate obtained from the winning MM precommitment model) correlated across the two experimental tasks. Subjects who precommitted more often in the effort task also did so in the delay task, *r_s_*_56_ = 0.346, *p* = 0.008. Consequently, the degree to which individuals profited from precommitment, quantified by the relative increase in achieved LRs in precommitment compared to standard trials, also correlated significantly across the two tasks, *r_s_*_56_ = 0.372, *p* = 0.004. In conclusion, participants' tendency to use precommitment, and therefore the individual-specific effectiveness of offering a precommitment option, generalized across the two investigated self-control contexts.

## 4. Discussion

We examined the effectiveness of precommitment in an effort-related and a delay-related choice task and assessed to what purpose participants employed precommitment, to overcome anticipated failures in willpower or to maximize their motivation for choosing the effort-/waiting-requiring LR option. Three main findings were obtained: first, participants used precommitment frequently and effectively in both tasks. Second, computational modelling showed that participants’ choices and precommitment decisions were more consistent with the motivation maximization hypothesis than the willpower hypothesis, indicating that participants used precommitment to optimize their motivation for choosing the effort-/waiting-requiring LR by extinguishing opportunity costs (i.e. as a self-motivational measure), rather than to prevent anticipated willpower failures (i.e. as a self-regulatory measure). Third, individuals' propensity to precommit correlated across the two tasks, suggesting that the tendency to use precommitment generalizes across self-control contexts.

Our main aim was to test the effectiveness of precommitment in the context of effort-requiring goals, providing an empirical basis for application of precommitment schemes in exercise and rehabilitation interventions. We found that precommitment is indeed effective in helping individuals achieve effort-requiring goals. In our paradigm, participants could precommit to an effort-requiring LR option by removing an effort-free SR option from their upcoming choice set. On average, participants used precommitment in 50% of possible instances and consequently achieved the large reward significantly more often in precommitment than in standard trials. Our results suggest that precommitment could be a promising strategy to increase exercise behaviour in insufficiently active individuals and help reduce the disease burden caused by the sedentary lifestyle of our modern society. A recent field study by Royer *et al*. [34] provides encouragement that this could work in real-life settings. In their study, company employees were first financially incentivized to use the company gym for one month and then given the voluntary option to wage some of the earned money on their continued use of the gym for another two months. Gym attendance of those who agreed to this precommitment exceeded that of those who did not by 25%. Precommitment could also be a fruitful way to increase training frequency in (neurological) rehabilitation patients. Hospitalized orthopaedic and stroke patients spend far too much time inactive (e.g. [35–37]) and fail to conduct self-directed training as regularly and intensively as recommend [3], and therefore may achieve suboptimal recovery (e.g. [38]). Precommitment implementations that reduce opportunity costs of self-directed rehabilitative training, such as blocking the bed during the daytime or restricting other activities, might help patients adhere better to exercise recommendations.

Our second aim was to determine to what purpose participants employed precommitment. A popular assumption is that precommitment is used to overcome anticipated willpower failures (e.g. [15,18,22,24]), where willpower is understood as costly [39] or resource-limited [40] top-down inhibition of action impulses. We found little evidence for such a self-regulatory use of precommitment. On our tasks, willpower failures would manifest in opt-outs of LR choices during the effort-execution/waiting phase (cf. [22]). Such instances were very sparse, indicating that our participants rarely experienced willpower failures. Consistently, model-derived estimates of wilful suppression of the SR during choice did not differ from those estimated for the subsequent effort-execution/waiting phases. Also, Bayesian comparison of the willpower and motivation maximization choice models revealed that participants’ choices between the effort-free/immediate SR and the effort-/waiting-requiring LR could be equally well explained by the less complex motivation maximization hypothesis, which formulates these choices as purely value-based decisions and postulates that precommitment is employed to increase the net motivational value of a target activity by reducing opportunity costs (cf. [9]). Moreover, observed rates of precommitment to the LR increased with a higher relative value of the LR option (compared to the SR option), that is to say, followed the value of precommitment as determined by the MM model. By contrast, the value of precommitment as determined by the WP precommitment model predicted the exact opposite pattern. Consequentially, the MM precommitment model had a better fit to observed precommitment decisions. Our observation that precommitment rates grow with the relative subjective value of the LR aligns with a simulation-based prediction by Kurth-Nelson & Redish [41] that probability of precommitment in intertemporal choices should increase with a higher larger-later to smaller-sooner reward ratio, and is broadly consistent with the suggestion that self-control is only imposed when its benefits outweigh its intrinsic costs (e.g. [39,42–44]). One question that might arise is why our motivation maximization hypothesis does not predict that precommitment would be used most often when it is most effective, i.e. when the difference between the subjective values of the LR and SR options is small, rather than when the relative subjective value of the LR is large? This would require a two-stage decision process in which the same valuation is used twice but with opposite behavioural consequences. Participants would first have to determine whether the subjective value of the effort/waiting- requiring LR option is higher than that of the effort-free/immediate SR option, whereby a larger difference would be stronger evidence for precommitment, and then assess the effect strength of precommitment, whereby a smaller difference would be stronger evidence for precommitment. Our data do not provide any support for such a complex process; rather, the observed precommitment rates showed the pattern predicted by our MM model, demonstrating that precommitment decisions can be described as a function of the integrated subjective value of choice options. Finally, one might wonder whether participants simply precommitted more often when the subjective value of the LR was higher because this corresponded to lower effort/waiting requirements, to which participants might be more willing to precommit to independently of reward value and integrated subjective value. We believe this to be unlikely because, if this was the case, then the effort-free/immediate SR option should arguably always have been judged more attractive.

Our finding that participants used precommitment to maximize their motivation, even when failures to choose the more advantageous option were not absolute, has important implications for real-life implementations: to date, trialed precommitment schemes focused primarily on complete failures to reach binary intervention targets, such as stop smoking [20], buy 5% more healthy groceries [21], lose one pound per week [45], or continue using the gym for two months [34]. Our results indicate that precommitment is effective as a *behaviour-optimization* strategy even in situations where people sometimes execute their health-relevant intentions, but do not always manage. Here, precommitment could serve to increase the frequency and intensity of the target activity. This is, for instance, the case in neurorehabilitation, where more training is proven to lead to a greater functional outcome, but where observed training frequency, intensity and duration often fall short of the recommended levels (see [3,46]). Similarly, based on the willpower hypothesis precommitment would be considered fruitful only for the willpower-weak, but our results demonstrate that precommitment is effective independently of willpower capacity. Finally, extant field studies all used financial penalties or loss of financial rewards as precommitment options and suffered from low acceptance rates (between 11% and 36%). Our findings suggest that precommitment schemes targeting opportunity costs should also be explored and might have higher acceptance rates.

We further found that individuals' propensity to accept precommitment correlated across the two tasks, indicating that a person's tendency to use precommitment generalizes across different types of self-control challenges. Future research might investigate whether stable trait-like differences exist for self-control/motivational strategies and how use of precommitment could be encouraged in the non-affine.

In conclusion, this study demonstrated that precommitment is an effective self-motivational and behaviour-optimization tool in the context of effort-requiring and waiting-requiring goals. Also, that this is true independently of willpower strain or failures and even when targets are only sometimes, but not always, failed. Our laboratory findings provide strong encouragement for the implementation of precommitment schemes in clinical prevention and rehabilitation programs in order to optimize exercise and other health-supporting behaviours.

## Supplementary Material

Supplementary Material

## Endnotes

1 Pilot testing identified 6000 ms as a sufficiently long execution phase for participants to perform the maximum level of required squeezes. This was corroborated by the current data: failure to reach the required number of squeezes occurred in only 0.7% of all trials in which subjects chose the LR option and did not subsequently opt-out to the SR, and only nine subjects experienced such an event at all, eight of which only once.

2 Responses on trials where subjects accepted precommitment were not included since only the LR was available during the choice phase in these trials, rendering Δ*V* = *SV*_LR_ and P(Choice LR) = 1.

3 Addition of the *b* parameter significantly increased goodness-of-fit compared to use of a single parameter model with only *θ*, without changing the results of the comparisons of the MM versus WP precommitment model.

## Data accessibility

Additional data available as part of the electronic supplementary material.
