# Supplementary material for: Conquering the inner couch potato: precommitment is an effective strategy to enhance motivation for effortful actions
Source: Philos Trans R Soc Lond B Biol Sci. 2018 Dec 31;374(1766):20180131. doi: 10.1098/rstb.2018.0131 (PMC6335452; doi:10.1098/rstb.2018.0131)
Supplement: Supplementary Material [file rstb20180131supp1.pdf]

Supplementary Material for Article

"Conquering the inner couch potato: Precommitment is an effective strategy to enhance motivation for effortful actions"

by

Bettina Studer[1,2], Carolin Koch[1], Stefan Knecht[1,2],Tobias Kalenscher[3]

1 Institute of Clinical Neuroscience and Medical Psychology, Medical Faculty, University of Dusseldorf, Dusseldorf, Germany

2 Mauritius Hospital Meerbusch, Meerbusch, Germany

3 Institute of Experimental Psychology, University of Dusseldorf, Dusseldorf, Germany,

Content

Supplementary Methods 3
Determination of maximum handgrip strength 3
Supplementary logistic regression analysis of LR Achievement 4
Statistical analysis of choice response times 4
Graphical representation of the choice model parameters (ε/κ, ι, γ) 5
Parameter recovery simulation 6
Supplementary Results 9
Comparison of MM an WP choice models on individual subject level 9
Complimentary analyses of LR Achievement and discounting parameters 11
Impact of required effort /delay does not differ between the two trial types 11
Time-on-task effects 12
Response times of precommitment decisions 13
Effort Task 13
Delay Task 14
Choice reaction times 14
Effort Task 14
Delay Task 15
Subgroup comparison of choice models 16
Effort Task 16
Delay Task 16
Supplementary References 17

Figure S1. The hand-held dynamometer. 3
Figure S2. Visualization of the parameters of the choice models 6
Figure S3. Exemplary results of the parameter recovery simulations 9
Figure S4. Effort Task: Subject-specific BIC scores of the choice models 10
Figure S5. Delay Task: Subject-specific BIC scores of the choice models 11
Figure S6. Response times during the pre-choice precommitment phase 14
Figure S7. Response times during the main choice phase 15

Supplementary Methods

Determination of maximum handgrip strength

A hand-held force dynamometer (Vernier Software & Technology, Beaverton, USA; see Figure S1A) was used in our effort task. To match subjective effort levels of choice options across subjects, individualized force levels (50% of the individual's maximum strength) were used in this task. Each individual's maximum handgrip strength was determined with a computerized pre-test, which consisted of four trials. The first trial (lasting 5000ms) primarily served to familiarize subject with the equipment and to get a first estimate of their strength. In this trial, participants were instructed to squeeze the dynamometer several times with as much force as they could. The (time course of the) produced force was visualized online on the computer screen (Figure S1B). In trials 2-4, participants were presented with a dynamic bar providing an online visualization of applied force and a target line, which they should try to exceed (Figure S1C). The target line and scale of the dynamic bar were adjusted in each trial so that the target line always represented the highest force recorded in the previous trial(s). Each trial lasted 3000ms seconds, and trials were separated by a 10-second ITI during which subjects were instructed to relax. The individuals' maximum strength was determined as the highest force recorded in any of these trials.

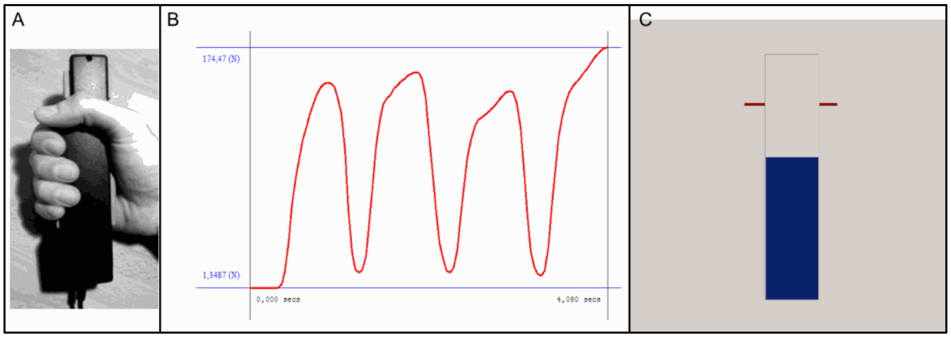

Figure S1. The hand-held dynamometer (A), and an exemplary first trial (B) and an exemplary main trial (C) from the pre-task determining each individual's maximum handgrip strength are displayed.

Supplementary logistic regression analysis of LR Achievement

Three extensions of the repeated measures logistic regression model of the outcome variable ‘Achievement of LR’ reported in the main text were tested in supplementary analyses. The first extended model assessed a potential interaction effect of trial type and delay/effort requirement interaction, and thus contained the two original predictors and the predictor ‘Trial Type x Waiting/Effort Requirement Interaction’.

The second and third extended models tested for potential time-on-task effects, because it has been argued that willpower is subject to depletion over time (e.g. 1, 2, 3). The second model did so by adding the linear predictor ‘Trial Number’ as a proxy for time on task to the original predictors ‘Trial Type’ (*precommitment* or *standard*) and ‘Waiting/Effort Requirement of the LR’. The third extended models checked for an effect of task order, as a coarser proxy for time on task, upon LR achievement. This logistic regression model included the categorical between-subject factor ‘Task Order’ in addition to the aforementioned two original predictors. For all three extended models, we tested the effects of these additional predictors and compared model fit of the extended models to that of the original model through obtained Corrected Quasi Likelihood under Independence Model Criterion (QICC). A better model fit is indicated by a lower QICC.

Finally, we also tested for an effect of task order upon the subject-specific effort and delay discounting parameters (ε and κ) obtained from fitting the MM choice model, through a t-test comparison of the participants who conducted the given task first versus second.

Statistical analysis of choice response times

As a supplement to the main statistical analyses reported in the manuscript, we also explored participants’ choice response times on the effort and delay tasks. For reaction times

during the pre-choice precommitment phase in *precommitment trials*, repeated measure analyses of variance (ANOVAs) with the within-subject factor Effort Level (2, 4, 6 squeezes) or Delay (4, 7, 10 sec) were calculated. For the main choice reaction times, repeated measure ANOVAs with the within-subject factors Trial Type (*precommitment* vs *standard trials*) and Effort Level (2, 4, 6 squeezes) or Delay (4, 7, 10 sec) were calculated. Note that we expected main choice reaction times to be shorter in *precommitment trials* because, in these trials, participants should have already processed and contemplated the two available choice options during the pre-choice phase. Greenhouse-Geisser corrections were applied to ANOVAs when homogeneity of variance was violated, and post-hoc t-tests were corrected for multiple comparisons by Sidak adjustment of p-values. All statistical tests are reported two-tailed, and alpha was set at .05.

Graphical representation of the choice model parameters (ε/κ, ι, γ)

Figure S2 provides a graphical simulation of the impact of variation in the parameters of the choice models, i.e. the discounting parameter ε, the wilful suppression parameter ι (WP choice model only), and the inverse temperature parameter γ, upon the model predicted net value of choosing the effort-requiring LR option (ΔV) and the predicted probability of choosing the LR option (P(Choice LR)). In each column, the isolated effect of variation in one parameter is shown. Note that the MM model does not include an ι parameter. This is numerically equivalent to $ι = 1$. The choice model of the effort task is visualized; parameter influences is qualitatively identical in the delay task.

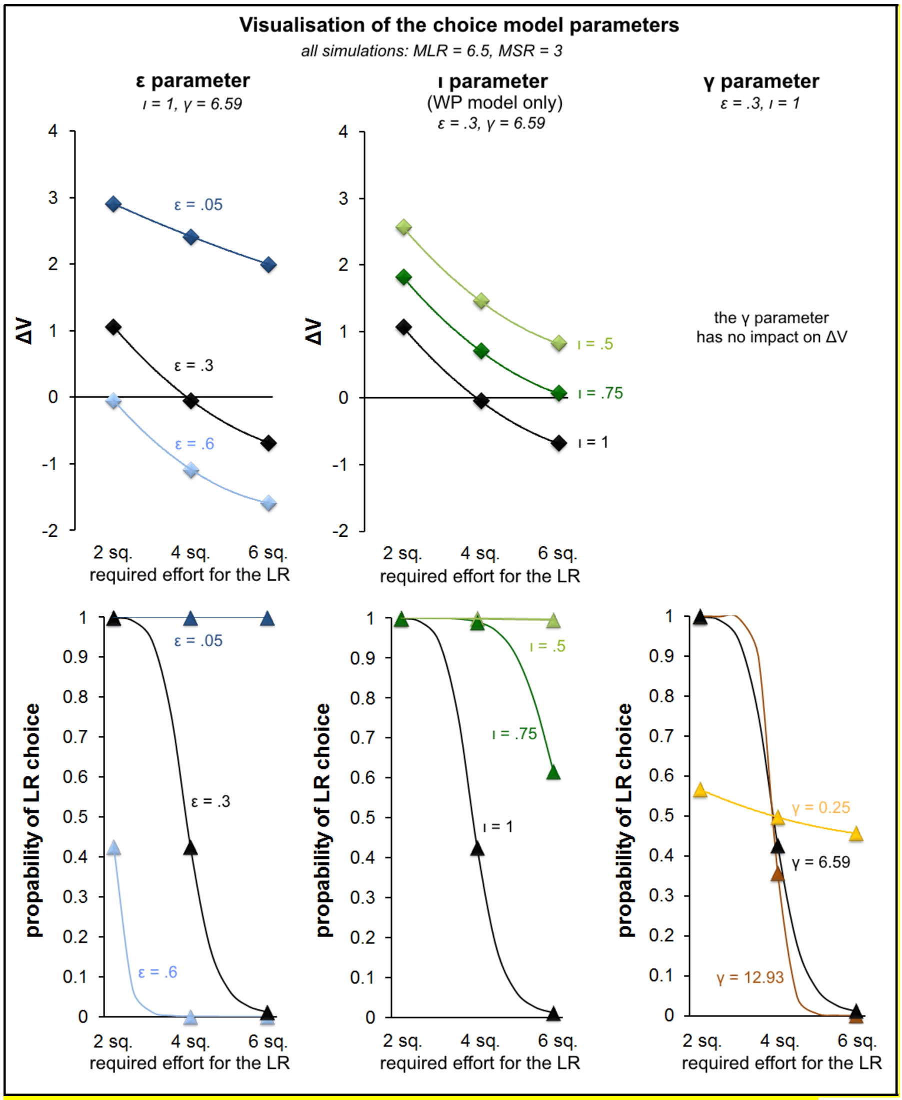

Figure S2. Visualization of the parameters of the choice models

Parameter recovery simulation

A parameter recovery simulation serves to assess the reliability and accuracy of the estimation of individual model parameters, given the model and data range, and involves generating synthetic data from the model (and inputted “true” parameters), fitting this synthetic data with the same model and techniques as the original data, and comparing the resulting parameter estimates to the inputted parameters (4). It can also reveal dependencies in parameter estimation, such that recovery of one parameter is adequate only within certain limits of another

parameter. We conducted parameter recovery simulations for all of our computational models to test for potential constellations where parameter estimates might be unreliable. In short, for each model we used the obtained parameters and recorded rating values of our participants to create 58 synthetic participants, computed 10 simulations of responses of these synthetic participants (60 SR vs LR choices in the case of the choice models, and 15 precommitment decisions in the case of the precommitment models, each equal numbers of each effort/waiting requirement level), fitted the simulated data with our models, and compared the mean values of the obtained 'recovered' parameters from the 10 simulations against the inputted parameters. By using the obtained parameter estimates of our subject in the synthetic data generation, we ensured that our synthetic participants match to and cover the full range of parameter constellations observed when fitting the models to the real data. In the case of WP choice models, we used obtained estimates of the κ and γ parameters only, and ran three sets of 10 simulations for each participant, one with $\iota = 0.3$ (very strong suppression), one with $\iota = 0.5$ (strong suppression) and one with $\iota = 0.7$ (moderate suppression). This allowed us to test whether this variation in the inputted suppression strength was replicated in the recovered parameter estimate.

The parameter recovery simulations showed adequate recovery of all parameters of the MM choice model, the MM precommitment model and the WP precommitment model, with correlations of $r \geq .8$ between inputted and (mean) recovered parameters estimates (see Figure S3A for an example), and no apparent dependencies between the recovery of the different parameters. In the recovery simulations of the WP choice model, we found that recovery of the ι parameter became unreliable for synthetic participants with very small discounting parameters, such that variation in the inputted ι did not result in a corresponding variation in the recovered ι (see Figure S3 B vs. C). This implies that participants who apply no discounting to the

effort/waiting-requiring LR options should be excluded from the choice model comparisons. The problem is that the true discounting parameters of our real participants are not known. Fortunately, the parameter recovery simulations also indicated that such cases with no or only very minimal discounting can be recognized by a complete lack of variance in the LR versus SR choices (always accepting the effort-/waiting requiring LR option). This pattern was not completely, but almost, exclusive to no-discounting cases. Thus, we used this proxy as an exclusion criterion, and recalculated the choice model comparisons for the remaining subsample of participants with (very likely) reliable parameter estimates. This resulted in subsample sizes of n=39 for the effort task, and n= 42 for the delay task (see pages 16 and 17 for results)

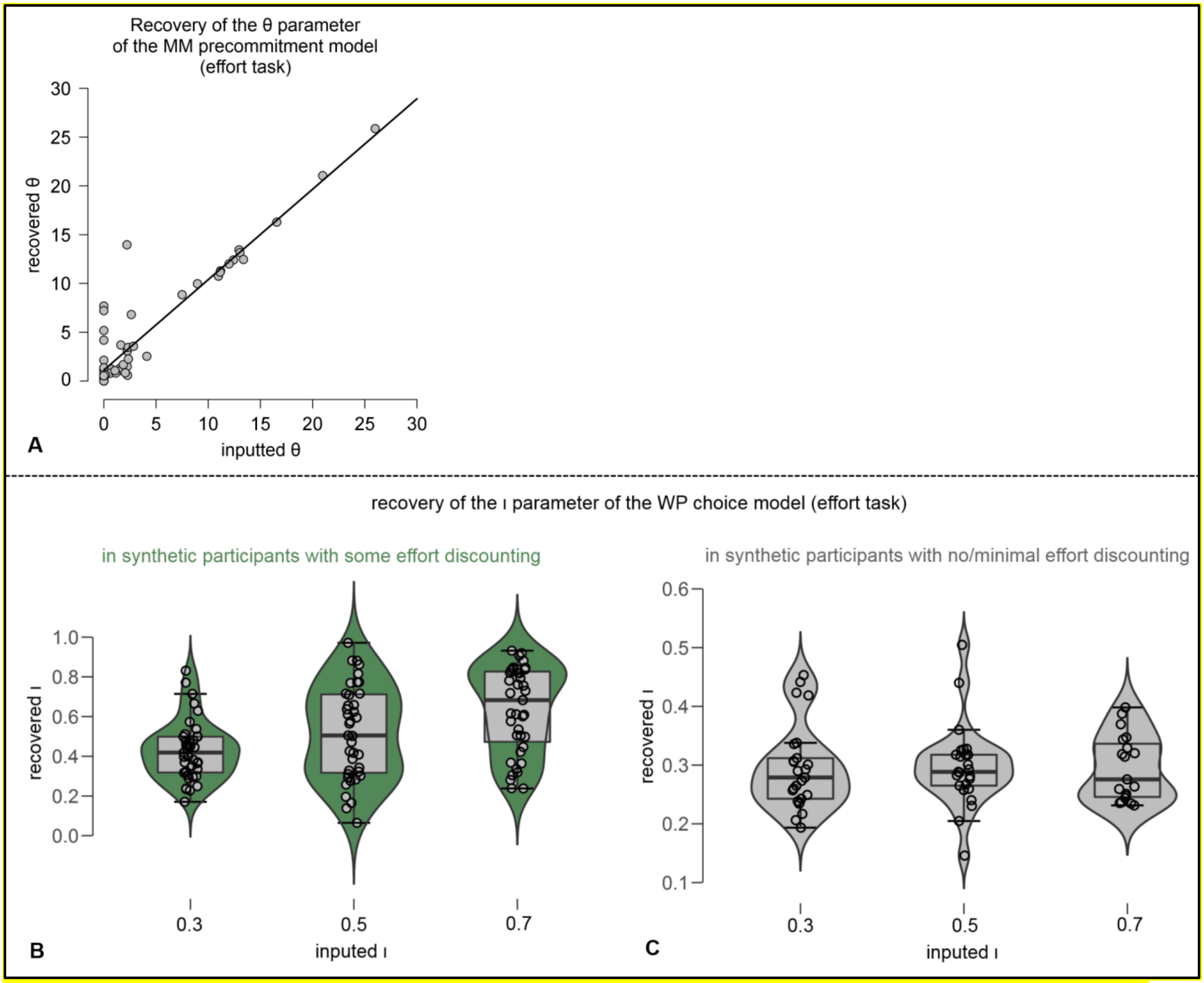

Figure S3. Exemplary results of parameter recovery simulations from the effort task. A: Good recovery of the θ parameter of the MM precommitment model. Each data point represents one synthetic participant. The x-axis shows the inputted θ, the y-axis the (mean) estimated θ in the recovery simulation, with $r(57)=.93$, $p < .001$. B+C: Recovery of the ι parameter of the WP choice model was adequate in participants with some effort discounting (B, note that the recovered ι increases with inputted ι values) but poor in participants with no/minimal effort discounting (C, note the lack of change in the recovered ι across the different inputted ι values). Each dot represents data from a synthetic participant, the violin shapes and box plots visualise the distribution of values in the subsamples.

Supplementary Results

Comparison of MM an WP choice models on individual subject level

The MM choice model outperformed the WP model not only on the group level but also on the level of individual subjects. For all 58 participants, the BIC scores for the MM model

were credibly smaller (i.e. absolute score difference > 2, (5)) than those of the WP model for both the modelling of choice on the effort task (Figure S4) and the modelling of choices on the delay task (see Figure S5). Obtained individual BIC scores differences ranged between -3.14 and -4.09 for the effort task and -2.80 and -4.09 for the delay task.

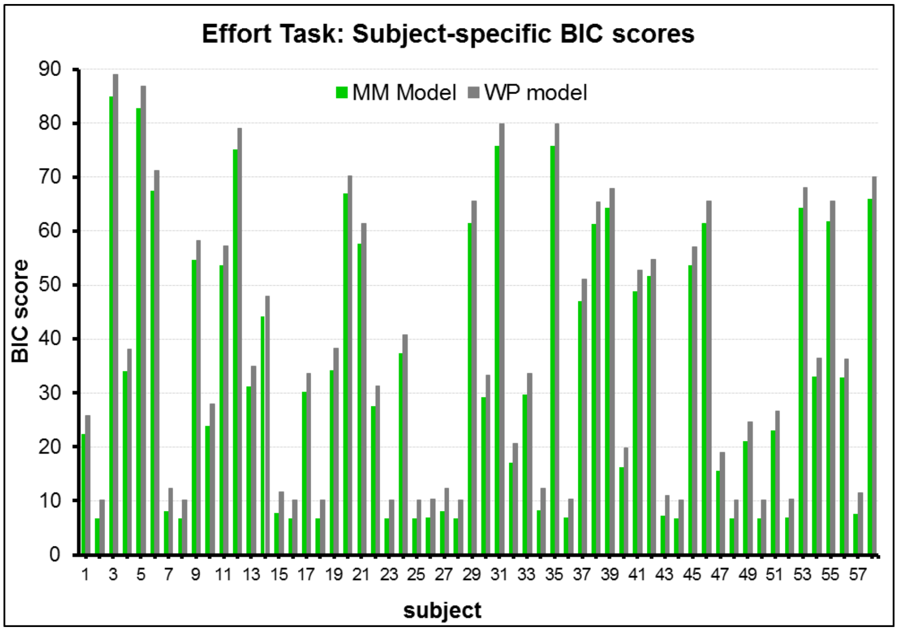

Figure S4. Effort Task: Subject-specific BIC scores for the MM choice model (green) and the WP choice model (grey). For all subject, BIC scores were credibly smaller for the MM choice model such that superiority of the MM choice model could be confirmed for each individual.

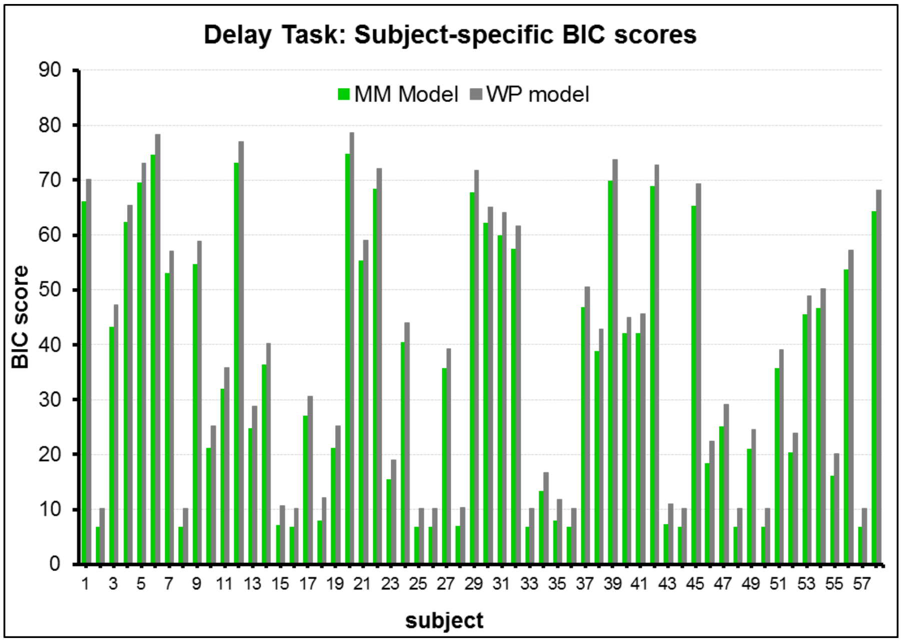

Figure S5. Delay Task: Subject-specific BIC scores for the MM choice model (green) and the WP choice model (grey). For all subject, BIC scores were credibly smaller for the MM choice model such that superiority of the MM model could be confirmed for each individual.

Complimentary analyses of LR Achievement and discounting parameters

Impact of required effort /delay does not differ between the two trial types

Our analyses yielded no evidence for an interaction effect of trial type and delay/effort requirement upon achievement of LRs. For both tasks, the interaction effect was non-significant, effort task: *Wald* $\chi^2(2) = 4.82$, $p = .282$, delay task: *Wald* $\chi^2(2) = 3.31$, $p = .19$, and QICCs for the extended logistic regression models (with the additional predictor 'Trial Type x Delay/Effort Requirement') were larger than those of the original model with the simple effects only (effort task: $QICC_{Extended} = 3361$, $QICC_{Original} = 3359$, delay task: $QICC_{Extended} = 3928$, $QICC_{Original} = 3926$), indicating a poorer fit. The effects of the two simple predictors, 'Trial Type' and 'Delay/Effort Requirement', remained qualitatively unchanged and were confirmed as significant (see main text for details).

Time-on-task effects

The second extended repeated measures logistic regression model tested whether achievement of LRs changed as a function of time-on-task through the predictor ‘Trial Number’. Indeed, a small, but statistically significant effect of the predictor ‘Trial Number’ was found for both tasks, such that probability of achieving the LR decreased with longer time-on-task, effort task: *Wald* $\chi^2(1) = 6.81$, $\beta(SE) = -.006\ (.002)$, $p = .009$, delay task: *Wald* $\chi^2(1) = 7.87$, $\beta(SE) = -.006\ (.002)$, $p = .005$. However, only for the delay task did this extended model also show a superior fit to the observed data than the original model ($QICC_{Extended} = 3921$, $QICC_{Original} = 3926$), whereas the opposite was true for the effort task ($QICC_{Extended} = 3414$, $QICC_{Original} = 3359$). The effects of the two main predictors again remained qualitatively unchanged and were confirmed as significant for both tasks (see main text for details).

The third extended repeated measures logistic model tested for an effect of task order, as a coarse index of a potential time on task effect. For the effort task, a tentative effect of task order emerged, *Wald* $X^2(1) = 2.950$, $p = .086$, β(SE) = -.62 (.361), such that the predicted average probability of LR achievement was ~10% higher for subjects who conducted this task first, $M_{Effort\ Task\ first} = .85$, SEM = .03, $M_{Effort\ Task\ second} = .76$, SEM = .05. The fit of this extended model was not superior to that the original model ($QICC_{Extended} = 3368$, $QICC_{Original} = 3359$). For the delay task, no systematic influence of task order was found, *Wald* $X^2(1) = 1.147$, $p = .284$, β(SE) = -.43 (.403).

Finally, we also tested for a potential task order effect upon effort and delay discounting parameters (ε and κ) obtained from the MM choice model. The effort discounting estimates were numerically lower, indicating stronger effort discounting, in subjects who conducted the effort task second (M = .261, SEM = .04) compared to those who conducted it first (M = .152, SEM

= .06), but this difference did not reach statistical significance, $t(56) = -1.503$, $p = .14$. For the delay discounting estimates, no systematic differences emerged between the two task subgroups, $t(56) = .076$, $p = .94$.

In summary, these analyses show a weak effect of time on task, such that achievement of large rewards decreased slightly with increasing trial number on both tasks. What might be the cause of this effect? Based on the proposition that willpower is a limited resource that depletes over time (e.g. 1, 2, 3), one might argue that this was caused by decreasing wilful suppression of SR choices. An alternative explanation is that subjective effort and delay costs increased with time-on-task, for instance, due to commencing muscle fatigue and boredom, respectively. Such increasing subjective effort and delay cost would, in turn, decrease the subjective value of the LR option and thereby the net motivation for choosing the delayed/effort-requiring LR option. Given that computational modelling of our data confirmed that participants' choices were closely determined by the difference in the subjective values of the SR and LR options (ΔV, see main text) and found no support for a need of wilful suppression of the SR option, we argue that this latter explanation is more plausible. Our observation that effort discounting tended to be higher when subjects performed the effort task second – albeit not at the level of statistical significance – also fits with this explanation.

Response times of precommitment decisions

Effort Task

On average, participants took 2690ms to indicate whether they wanted to precommit or not on in *precommitment trials* (SD=1441ms). Average pre-choice response times did not

significantly vary across different levels of required effort for the large reward option, *F*(1,57) = .386, *p* = .682, η^2 = .014, see Figure S6A.

Delay Task

Subjects took 2446ms on average to indicate whether they wanted to precommit or not in *precommitment trials* (SD = 1155ms). In this task, average pre-choice response times were systematically and significantly influenced by the delay of the large reward option, *F*(1,57) = 6.80, *p* = .002, η^2 = .107, such that response times were longer in trials where the delay of the large reward was 7 seconds than in trials were delay was shorter (4 sec, p < .061) or longer (10 sec, p < .006), see Figure S6B.

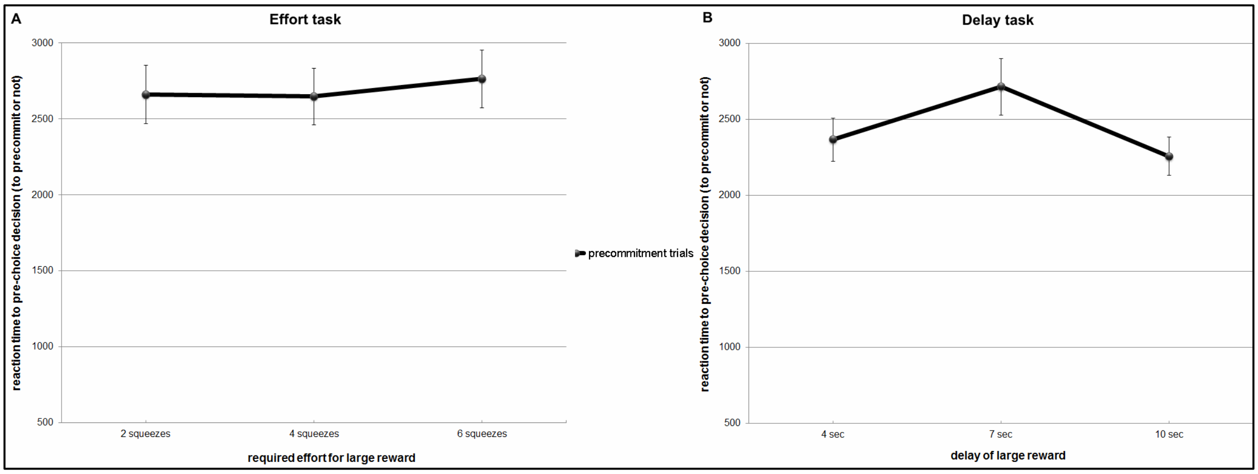

Figure S6. Response times during the pre-choice precommitment phase in the effort task (A) and in the delay task (B). Error bars represent SEM.

Choice reaction times

Effort Task

As expected, participants' response times in the main choice period were significantly higher in *standard trials* (M = 1829ms, SD = 1033ms) compared to *precommitment trials* (M=948ms, SD=886ms), *F*(1,57) = 58.099, *p* < .001, η^2 = .505. Furthermore, choice response times showed a tendency to increase with increasing effort requirements for the large reward,

F(2,114) = 2.376, *p* = .098, η^2 = .040, see Figure S7A. The ANOVA yielded no significant Trial Type x Effort Level interaction effect, *F*(2,114) = .048, *p* = .953, η^2 = .001.

Delay Task

Again, participants responded significantly slower in *standard trials* (M=1829ms, SD=1033ms) compared to *precommitment trials* (M=948ms, SD=886ms), *F*(1,57) = 57.326, *p* < .001, η^2 = .501. The ANOVA further revealed a marginally significant Trial Type x Delay Level interaction effect, *F*(2,114) =2.51, *p* = .086, η^2 = .042, which we investigated further with separate repeated measures ANOVAs of *precommitment* and *standard trials*. As can be seen in Figure S7B, choice response times increased linearly with increasing delay of the large reward in *standard trials*, *F*(1.77,100.72) =3.635, *p* = .029, η^2 = .060, but did not systematically vary as a function of delay of the large reward option in *precommitment trials*, *F*(1.82,103.72) =1.444, *p* = .241, η^2 = .025.

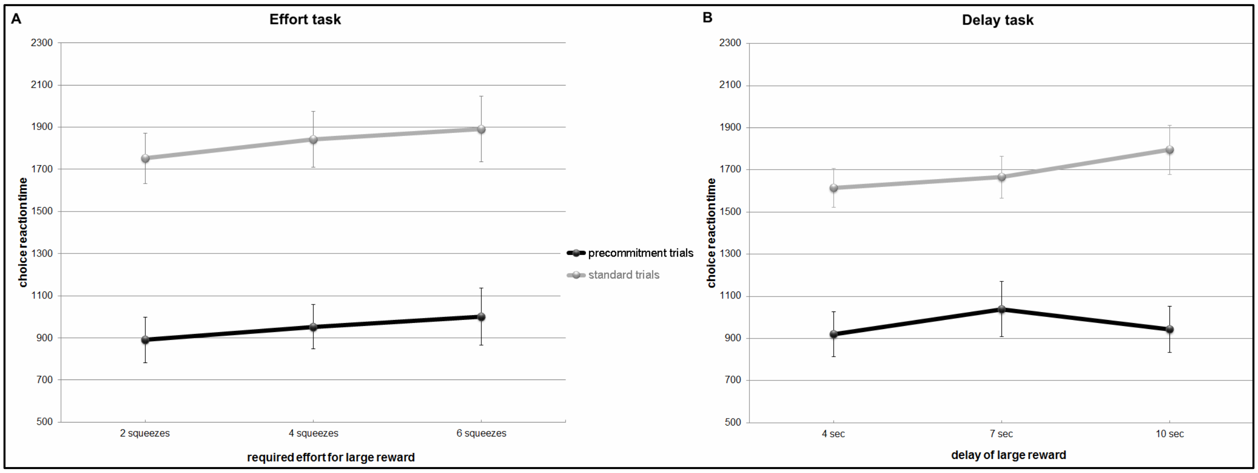

Figure S7. Response times during the main choice phase in the effort task (A) and in the delay task (B). Error bars represent SEM.

Subgroup comparison of choice models

Effort Task

The results of the MM versus WP choice model comparison conducted in participants with some variance in their LR choices only (n=39) were qualitatively identical to those of the entire sample reported in the main manuscript: The MM choice model strongly outperformed the WP choice model in the Bayesian model comparison, both on the group level ($BIC_{MM\ model}$ = 1839, $BIC_{WP\ model}$ = 1985) and for each individual participant (Δ individual BIC scores: M = -3.74, Range [-3.14, -4.09]). The average pseudoR^2s of the two models were identical and showed good fit (both models: M=.40, SEM =.05). Thus, the observed choices of participants without precommitment were equally well predicted by both models, but more parsimoniously explained by the less complex MM model. Furthermore, and again as was the case for the full sample analysis, WP model-derived estimates of ι_{reward} (M = .61, SEM =.057) were not systematically smaller than estimates of ι_{choice} (M = .60, SEM = .055), $t(38) = .160$, $p = .873$, contrary to the assumption of the willpower hypothesis that wilful suppression of the SR would sometimes fail during the effort execution period.

Delay Task

For the delay task, the results of the MM versus WP choice model comparison conducted in participants with some variance in their LR choices only (n=42) were also qualitatively identical to those of the entire sample reported in the main manuscript and those reported for the effort task above. Again, the MM choice model outperformed the WP choice model in the Bayesian model comparison, due to its lower complexity and thus lower BIC scores, $BIC_{MM\ model}$ = 1931, $BIC_{WP\ model}$ = 2088, Δ individual BIC scores: M = -3.74, Range [-3.14, -4.09], while fitting equally well to participants LR choice, as reflected in identical average pseudoR^2s (both

models: M=.43, SEM =.04). And again, the WP models assumption that wilful suppression would sometimes fail during the waiting period was not confirmed: the WP model-derived estimates of ι_{reward} (M = .46, SEM =.054) were not systematically smaller than estimates of ι_{choice} (M = .48, SEM = .055), $t(41) = -.56$, $p = .579$.

Supplementary References

1. Muraven M, Baumeister RF. Self-regulation and depletion of limited resources: Does self-control resemble a muscle? *Psychol Bull*. 2000;126(2):247.
2. Baumeister RF, Vohs KD, Tice DM. The strength model of self-control. *Curr Dir Psychol Sci*. 2007;16(6):351-5.
3. Gailliot MT, Baumeister RF, DeWall CN, Maner JK, Plant EA, Tice DM, et al. Self-control relies on glucose as a limited energy source: willpower is more than a metaphor. *J Pers Soc Psychol*. 2007;92(2):325.
4. Heathcote A, Brown SD, Wagenmakers E-J. An introduction to good practices in cognitive modeling. An introduction to model-based cognitive neuroscience: Springer; 2015. p. 25-48.
5. Schwarz G. Estimating the Dimension of a Model. *Ann Statist*. 1978;6(2):461-4.
